# Escaping alveolar macrophage endosomal retention explains massive expansion of SARS-CoV-2 delta variant

**DOI:** 10.1038/s41392-021-00845-4

**Published:** 2021-12-17

**Authors:** Zhenfeng Wang, Yabo Zhou, Linlin Bao, Dan Li, Jiadi Lv, Dianheng Wang, Shunshun Li, Wei-Min Tong, Jiangning Liu, Chuan Qin, Bo Huang

**Affiliations:** 1 Department of Immunology & National Key Laboratory of Medical Molecular Biology, Institute of Basic Medical Sciences, Chinese Academy of Medical Sciences (CAMS) & Peking Union Medical College, 100005 Beijing, China; 2 NHC Key Laboratory of Human Disease Comparative Medicine, Beijing Key Laboratory for Animal Models of Emerging and Remerging Infectious Diseases, Institute of Laboratory Animal Science, CAMS and Comparative Medicine Center, Peking Union Medical College, Beijing, China; 3 Department of Pathology, Institute of Basic Medical Sciences, CAMS and Peking Union Medical College, Beijing, China; 4 Department of Biochemistry & Molecular Biology, Tongji Medical College, Huazhong University of Science & Technology, 430030 Wuhan, China

**Keywords:** Infection, Infectious diseases, Innate immune cells

**Dear Editor**,

Genetic variant Delta (B.1.617.2) of severe acute respiratory syndrome coronavirus 2 (SARS-CoV-2), which possesses a remarkable ability to transmit and spread, is currently becoming predominant worldwide. Despite its great harm to human beings, how the Delta variant with T478K, P681R and L452R mutations achieves its ultrafast spread remains elusive. Entry of SARS-CoV-2 into host cells is mediated by a rapid enzymatic hydrolysis. Upon the binding of the SARS-CoV-2 surface spike protein to its receptor angiotensin-converting enzyme 2 (ACE2), cellular membrane enzyme TMPRSS2 directly cuts spike protein S2 subunit, allowing the viral and cellular membrane fusion and release of viral RNA into the cytoplasm. While this TMPRSS2-mediated entry is highly efficient, certain immune cell types such as macrophages can use phago/endocytosis rather than ACE2-dependent way to take up the virus.^1^ Upon endocytosis, endosomal protease cathepsin L (CTSL) cleaves spike protein S1 subunit, leading to the viral and endosomal membrane fusion and release of viral RNA into the cytoplasm. Like other lysosomal cathepsin members,^2^ the activity of endosomal cathepsin L relies on a low pH, which favors the protonation of substrate molecules with positive charge. Notably, either T478K, P681R or L452R mutation promotes spike protein protonation with positive charge, due to the increase of amino group from lysine (K) or arginine (R), promoting us to assume that Delta variant enhances endosomal spike protein cleavage by CTSL via the enhanced protonation.

A typical clinical symptom of SARS-CoV-2 infection is the dry cough, which hints that the virus initially invades the alveoli where abundant alveolar macrophages (AMs) readily respond to viral particles and the released interferons may stimulate the ACE2 upregulation and mucin production.^3^ Previously, we found that classically activated M1 AMs have a more acidic endosomal pH than alternatively activated M2 AMs, leading to M2 AMs possessing the ability to degrade and limit SARS-CoV-2 spread.^1^ This may explain a large number of infected people without symptom or just with mild symptom, considering human AMs are more like a M2 phenotype.^4^ Based on these analyses, we hypothesize that Delta variant use autoprotonation-biased spike protein to escape the endosomal retention in AMs for its ultrafast spread.

To test the hypothesis, we incubated S protein from different variants (Supplementary Fig. S1) with CTSL in vitro. We found that under an acidic condition of pH 6.0, CTSL moderately cleaved S protein with D614 (WT) or D614G, weakly cleaved Beta or Gamma S protein, but strongly cleaved Delta S protein (Fig. 1a and Supplementary Fig. S2a). When we increased the pH to 6.5, we found that CTSL was only able to cleave Delta S protein (Fig. 1a and Supplementary Fig. S2a). Further silver staining showed that CTSL mainly cleaved Delta S protein into 130 and 70 kDa two bands (Fig. 1b), suggesting that mutation to basic amino acids might increase the sensitivity of S protein to CTSL. However, the increase of pH to 6.8 resulted in the loss of CTSL enzymatic activity to cleave Delta S protein (Supplementary Fig. S2b). To validate these results in AMs, we infected AMs with different pseudo-type SARS-CoV-2 variants. Given the fact that once the S protein was cleaved by CTSL in endosomes, the exposed S1 domain can be recognized by anti-S1 antibody, we performed the western blot using the antibody. As expected, we found that S protein was highly efficiently cleaved by CTSL in pseudo-Delta-infected macrophages but not in other pseudo-variant-infected macrophages (Fig. 1c and Supplementary Fig. S2c). Therefore, Delta variant could enhance the cleavage of S protein by CTSL upon the uptake into the endosomes of AMs. Indeed, the use of either CTSL inhibitors or siRNA markedly inhibited the cleavage of Delta S protein in AMs (Fig. 1d–f and Supplementary Fig. S2d, e).

**Fig. 1:**
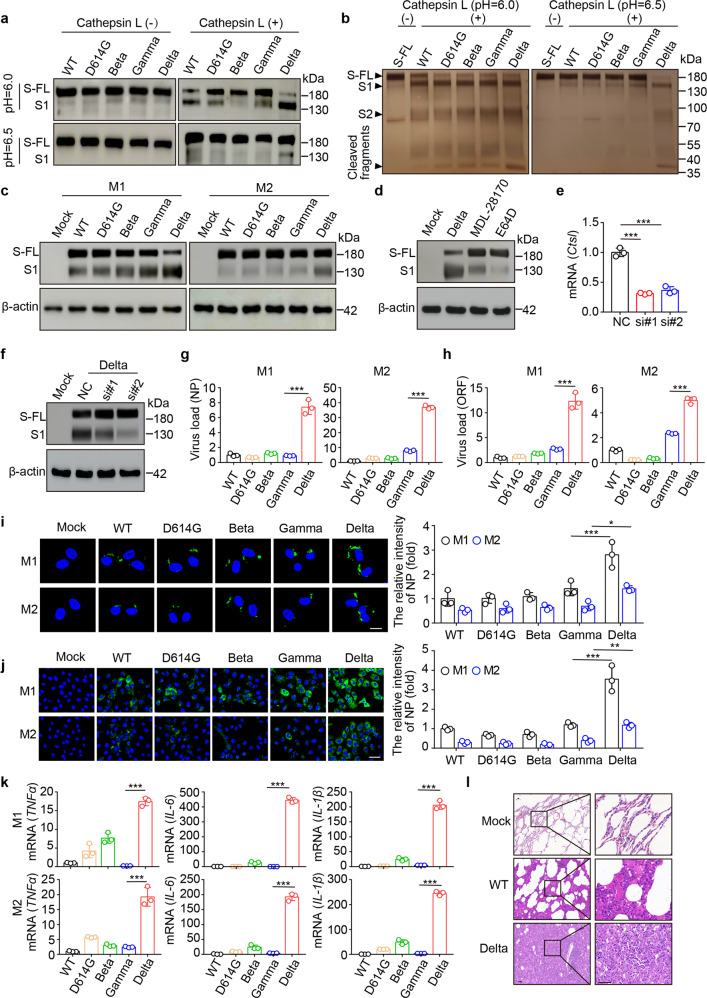
Delta variant usurps AMs for spread by evading endosomal retention. **a** Comparison of CTSL cleavage efficiency among different variants. The cleavage of spike protein (1 μg) by CTSL (20 ng) at 37 °C for 5 min is visualized by western blotting with anti-S1 antibody. The full-length (FL) S protein and cleaved S1 fragment were indicated. **b** The same as (**a**), except that the cleavage of spike was determined by silver staining. Black arrow heads indicate spike protein bands. **c** Different pseudoviruses of SARS-CoV-2 variants were incubated with AMs for 30 min, and then the cell lysates were detected by western blotting. **d** The same as (**c**), except that M1 was pretreated with CTSL inhibitors MDL-28170 (10 μM) or E64D (20 μM) for 2 h. **e** The efficiency of knockdown of *Ctsl* was detected by real time PCR. **f** The cleavage of spike in *Ctsl* knockdown M1 AMs was verified by western blotting. **g**–**i** AMs isolated from ICR mice were infected with SARS-CoV-2 WT or other variants for 30 min. The cells were washed to remove the extracellular viruses and re-cultured for another 4 h. The viral load was analyzed by qPCR (**g**, **h**) or immunostaining of NP (**i**). Scale bar, 10 μm. **j** The same as (**g**), except that the supernatants at 4 h were collected to infect Vero E6 cells for 24 h. Cells were stained with anti-NP antibody. Scale bar, 30 μm. **k** The same as (**g**), except that the mRNA of *TNFα*, *IL-1β* or *IL-6* was measured. **l** The pathological changes of lung tissues infected with SARS-CoV-2 WT or Delta variant were analyzed by H&E staining. Scale bar, 50 μm. The data are presented as mean ± SD. *p < 0.05, **p < 0.01, ***p < 0.001, by one-way ANOVA (**e**, **g**–**k**)

CTSL cleavage facilitates the release of viral RNA into the cytoplasm for replication. Consistently, following the infection of M1 or M2 AMs with different variants, NP protein, a structural protein which is critical for the assembly of the nucleocapsid and the release of progeny of SARS-CoV-2 particles,^1^ was highly expressed in Delta-infected macrophages, but lowly expressed in other variants-infected M1 and M2 AMs, as evidenced by real time PCR (Fig. 1g, h) and immunostaining (Fig. 1i). In line with this result, the percentage of Delta-infected AMs was much higher than other variant-infected AMs (Supplementary Fig. S2f). Following 30 min incubation of SARS-CoV-2 with M1 or M2 AMs, we washed and re-cultured the cells in the virus-free medium for 4 h. The supernatants were used to treat Vero E6 cells. The expression levels of NP protein were markedly different, highly in Delta supernatant-treated cells but lowly in other variant supernatants-treated cells (Fig. 1j). Thus, Delta variant can actively replicate in and be released from both M1 and M2 AMs.

SARS-CoV-2-infected macrophages may upregulate pro-inflammatory cytokines, which are thought to exacerbate the pathogenesis of SARS-CoV-2 infection. In line with this, Delta variant much more strongly stimulated both M1 and M2 AMs to upregulate the expression of TNF-α, IL-1β and IL-6 than other variants (Fig. 1k and Supplementary Fig. S2g). Consistently, the H&E staining of SARS-CoV-2-infected macaque lungs showed that Delta variant infection caused more peri-bronchial and peri-vascular inflammatory cell infiltration and an enhanced pathological damage, compared to the WT strain-infected lungs (Fig. 1l).

In summary, unlike previous S protein mutations such as K417N, N501Y or K417T, which do not increase the amino group (-NH3^+^), Delta S protein mutation (T478K, P681R and L452R) generates more amino groups (Supplementary Fig. S1), leading to the enhanced protonation and sensitivity to hydrolytic enzymes. While such protonated mutations have been reported to favor the furin cleavage which may facilitate the ACE2-mediated entry of the virus into epithelial cells,^5^ we think that these mutations-facilitated viral RNA release from the endosomal retention of M2 AMs might be more biologically significant, considering that AMs are the primary infected cells following SARS-CoV-2 entry into the alveoli. Although Delta variant may use the mutated S protein to facilitate the furin activity, thus promoting the infection of alveolar epithelial cells, this variant prefers the endocytosis for its entry into AMs. Our previous studies have demonstrated that SARS-CoV-2 can usurp more acidic endosomes of M1 AMs for viral escape, proliferation and spread; but a more basic endosome in M2 AMs is likely to detain the virus and limit its spread.^1^ Thus, the general presence of M2 AMs in normal alveoli of human beings^4^ and the relatively basic endosomal pH^1^ may explain why many SARS-CoV-2 infected people have no clinical symptom or just have mild symptoms. Unfortunately, Delta variant acquires basic amino acid mutations, which break the limitation from more alkaline endosomal pH and lead to viral replication in M2 AMs, thus achieving an ultrafast spread in populations. In addition, our findings might also explain why vaccines are less protective against Delta infection but still reduce severity of illness, hospitalization, and death. Vaccine-induced anti-S protein antibody binds to Delta variant S protein, which can be taken up by AMs through FcR-mediated endocytosis. However, CTSL is able to catalyze mutated S protein in endosomes, allowing the entry of Delta RNA into the cytosol and subsequent Delta RNA replication and new viral spread. Notwithstanding this, vaccine-induced anti-S antibody might still maintain the ability to bind to Delta mutated S protein, thus interfering with the interaction with ACE2, preventing Delta viral particles from infecting alveolar epithelial cells, and reducing the severity of illness.

## Supplementary information

Supplementary information

## Data Availability

All data needed to evaluate the conclusions in the paper are present in the paper or the Supplementary Materials. Materials described in the study are either commercially available or on request from the corresponding author.

## References

[1] Lv J (2021). Distinct uptake, amplification, and release of SARS-CoV-2 by M1 and M2 alveolar macrophages. Cell Disco..

[2] Willenbrock F, Brocklehurst K (1986). Chemical evidence for the pH-dependent control of ion-pair geometry in cathepsin B. Benzofuroxan as a reactivity probe sensitive to differences in the mutual disposition of the thiolate and imidazolium components of cysteine proteinase catalytic sites. Biochem J..

[3] Huang B (2021). Mucins produced by type II pneumocyte: culprits in SARS-CoV-2 pathogenesis. Cell. Mol. Immunol..

[4] Desch AN (2016). Flow Cytometric Analysis of Mononuclear Phagocytes in Nondiseased Human Lung and Lung-Draining Lymph Nodes. Am. J. Respir. Crit. Care Med..

[5] Liu, Y. et al. Delta spike P681R mutation enhances SARS-CoV-2 fitness over Alpha variant. Preprint at *bioRxiv*10.1101/2021.08.12.456173 (2021). 10.1016/j.celrep.2022.110829 PMC9050581 35550680

