# Supplementary material for: Escaping alveolar macrophage endosomal retention explains massive expansion of SARS-CoV-2 delta variant
Source: Signal Transduct Target Ther. 2021 Dec 17;6:431. doi: 10.1038/s41392-021-00845-4 (PMC8679569; doi:10.1038/s41392-021-00845-4)
Supplement: Supplementary file 1 — Supplementary information [file 41392_2021_845_MOESM1_ESM.docx]

Supplementary Materials for

Escaping alveolar macrophage endosomal retention explains massive expansion of SARS-CoV-2 Delta Variant

Zhenfeng Wang^1,5^, Yabo Zhou^1,5^, Linlin Bao^2^, Dan Li^2^, Jiadi Lv^1^, Dianheng Wang^1^, Shunshun Li^1^, Wei-Min Tong^3^, Jiangning Liu^2^, Chuan Qin^2*^, Bo Huang^1,4,6*^

^1^Department of Immunology & National Key Laboratory of Medical Molecular Biology, Institute of Basic Medical Sciences, Chinese Academy of Medical Sciences (CAMS) & Peking Union Medical College, Beijing 100005, China

^2^NHC Key Laboratory of Human Disease Comparative Medicine, Beijing Key Laboratory for Animal Models of Emerging and Remerging Infectious Diseases, Institute of Laboratory Animal Science, CAMS and Comparative Medicine Center, Peking Union Medical College, Beijing, China.

^3^Department of Pathology, Institute of Basic Medical Sciences, CAMS and Peking Union Medical College, Beijing, China

^4^Department of Biochemistry & Molecular Biology, Tongji Medical College, Huazhong University of Science & Technology, Wuhan 430030, China

^5^These authors contributed equally

^6^Lead Contact

*Corresponding author: [tjhuangbo@hotmail.com](mailto:tjhuangbo@hotmail.com), [qinchuan@pumc.edu.cn](mailto:qinchuan@pumc.edu.cn)

**This PDF file includes:**

Figures. S1 and S2

Materials and Methods

**
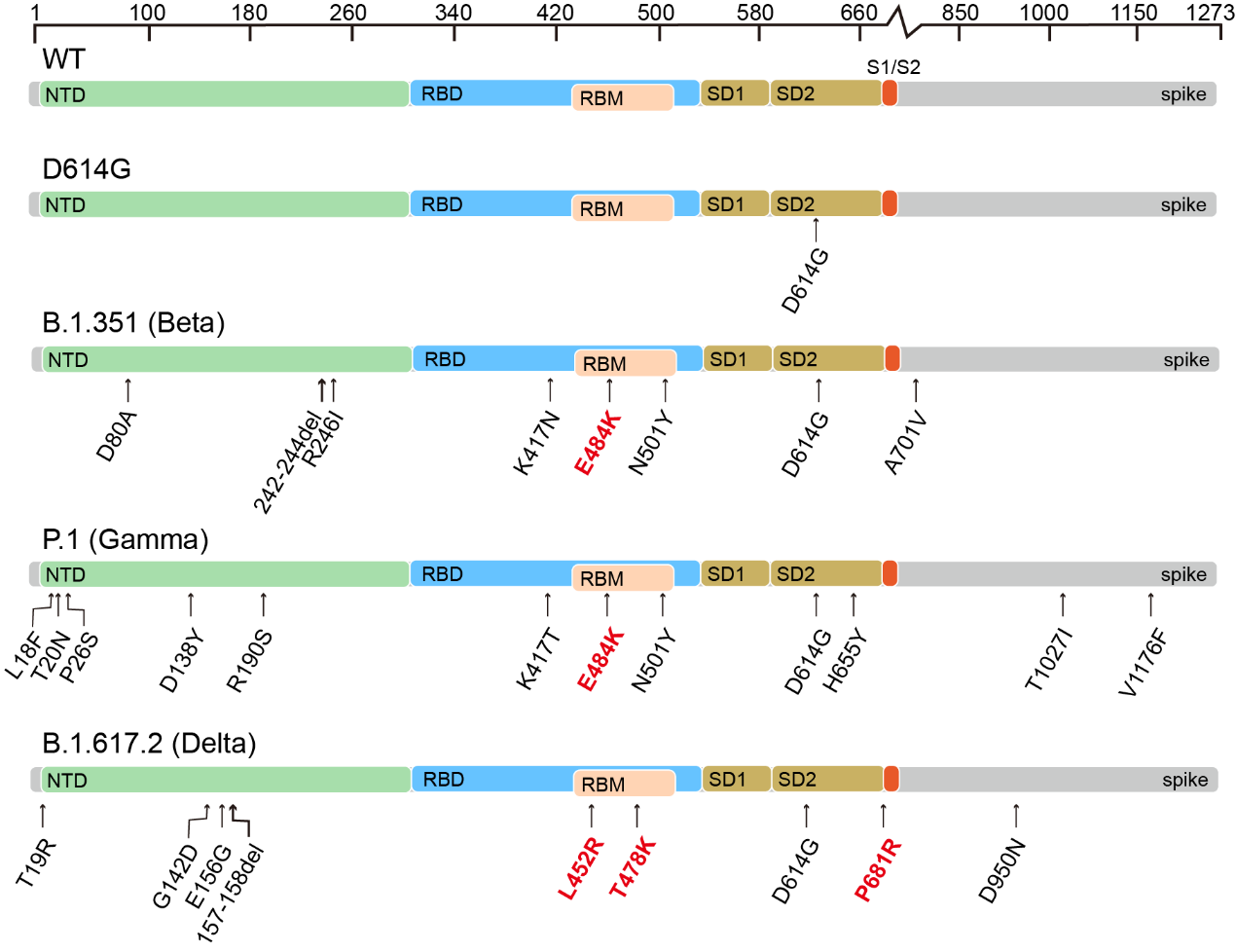
**

**Fig. S1 |** **The schematic diagram of SARS-CoV-2 variants.** Amino acid mutations are indicated compared to wild-type sequence. The red indicates basic amino acid mutation. NTD, N-terminal domain; RBD, receptor-binding domain; RBM, receptor-binding motif; SD1/SD2, subdomains.


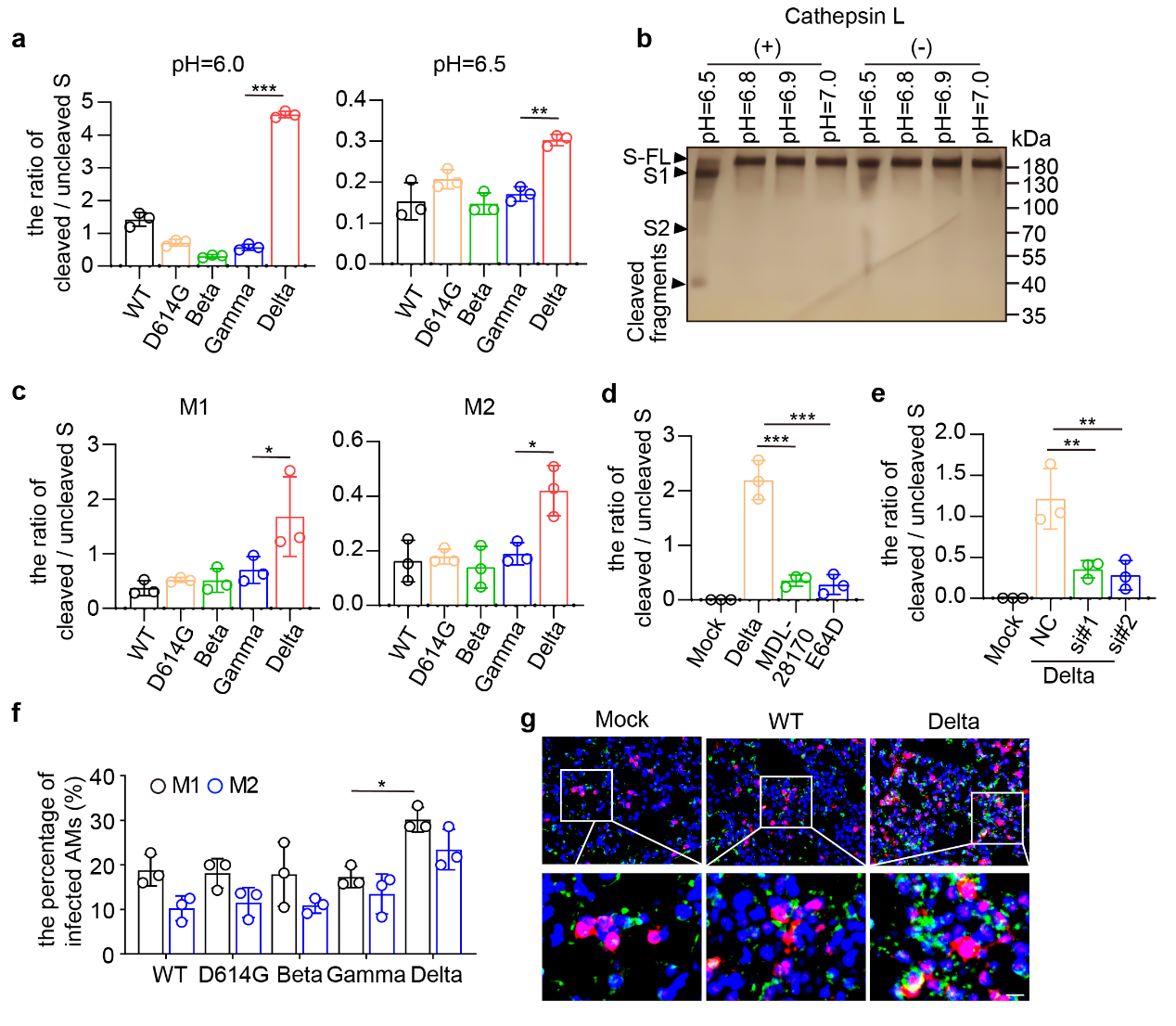


**Fig. S2 | Delta variants use CTSL to spread in macrophages. a, c, d, e** The statistical analysis of gray for fig.1a, 1c, 1d and 1f, respectively. **b,** Delta spike protein (1 μg) were incubated with CTSL (20 ng) at 37℃ for 5 min, the cleavage of spike was determined by silver staining. Black arrow heads indicate spike protein bands. **f,** AMs isolated from ICR mice were infected with SARS-CoV-2 WT or other variants for 30min. The cells were washed to remove the extracellular viruses and re-cultured for another 4 h. The percentage of positive immunostaining of NP was analyzed. **g,** hACE2-transgenic mice were infected with 1×10^5^ TCID_50_ SARS-CoV-2 WT or Delta for 5 days, then the lung tissues were fixed for immunofluorescence staining (red: anti-TNFα; green: anti-IL-6). Scale bar, 10 μm. The data are presented as mean ± SD. * p<0.05, ** p<0.01, *** p<0.001, by one-way ANOVA (a, c, d-f ).

**Materials and Methods**

**Animals and Cell lines**

Female ICR mice were purchased from the Center of Medical Experimental Animals of the Chinese Academy of Medical Science (Beijing, China). Chinese-origin rhesus macaques (3-4 years old) were obtained from the Institute of Laboratory Animal Science, Peking Union Medical College. Animals studies involving SARS-CoV-2 were performed in an animal biosafety level 3 (BASL3) facility using HEPA-filtered isolators and the procedures were approved by the Institutional Animal Care and Use Committee of the Institute of Laboratory Animal Science, Peking Union Medical College (BLL20001). Monkey kidney Vero E6 cell line was obtained from the Cell Resource Centre of Peking Union Medical College (Beijing, China) and cultured in DMEM medium (Gibco, Cat.: C11995500BT) with 10% FBS (Gibco, Cat.: 10099-141).

**Isolation of primary alveolar macrophages**

Primary alveolar macrophages (AMs) were isolated as described previously^5^. Briefly, the mice were anesthetized immediately prior to lavage and the trachea was dissected. Lungs were lavaged five times with 1 ml PBS and the retained BALF was centrifuged at 600 × g for 5 min at 4 °C. The pellet was resuspended in RPMI 1640 (Gibco, Cat.: C11875500BT) and harvested in a culture plate. The primary AMs were polarized to M1 by IFN-γ (Peprotech, Cat.: 315-05, 20 ng/mL) and LPS (Sigma, Cat.: L2630, 100 ng/mL) or M2 by IL-4 (Peprotech, Cat.: 214-14, 20 ng/mL) for 24 h.

**Proteolytic reactions *in vitro***

Recombinant 2019-nCoV (P.1) S-trimer Protein (DRA157), Recombinant 2019-nCoV (501Y.V2) S-trimer Protein (DRA153), Recombinant,2019-nCoV (B.1.617.2) S-trimer Protein (DRA168), Recombinant 2019-nCoV(D614G) S-trimer Protein (DRA59), Recombinant 2019-nCoV (WT) S-trimer Protein (DRA49), and cathepsin L(C401) were purchased from Novoprotein. To assay cleavage of spike by cathepsin L *in vitro*, 1 μg purified spike protein (wild-type, D614G, Beta, Gamma, Delta) were incubated with 20 ng cathepsin L at 37 °C for 5 min. The reactions were carried out in a buffer containing 50 mM MES (M8010, Solarbio, China), 5 mM DTT (D8220, Solarbio, China), 1 mM EDTA (E8040, Solarbio, China) adjusted to pH 6.0 or 6.5. Following the incubation, 5 μL 6 × SDS sample loading buffer was added to the reaction mixture and heated to 95 °C for 5 min. The samples were detected by silver staining (Beyotime Biotechnology, P0017S, China) or western blotting.

**Pseudovirus or virus infection with AMs**

For pseudovirus (Sino Biological, China) infection with AMs, SARS-CoV-2 Delta (B.1.617.2 XCV08), SARS-CoV-2 (501Y.V2, XCV05), SARS-CoV-2 (P1, XCV06), SARS-CoV-2 (D614G, XCV02) and SARS-CoV-2 (WT, XCV01) were incubated with AMs (5 × 10^4^) for 30 min, and then the cell lysates were detected by western blotting. For virus infection with AMs, the different variants (5 × 10^4^ TCID50) were incubated with AMs (5 × 10^4^) for 30 min, then virus was removed and cells were re-cultured for another 4 hr.

**Western blotting**

The protein was run on a SDS-PAGE gel and transferred to nitrocellulose. Nitrocellulose membranes were blocked in 5% bovine serum albumin (Sigma, Cat.: B2064) and probed with antibodies overnight: anti-spike (Sino Biological, Cat: 40592-T62; 1:1000) and anti-β-actin (Cell signaling technology, Cat.: 3700S; 1:1,000). Secondary antibodies conjugated to horseradish peroxidase were followed by enhanced chemiluminescence (Thermo fisher, Cat.: 32209).

**Real-time PCR**

Total RNA was extracted from cells using Trizol (Invitrogen, Cat.: 15596026) and was transcribed to cDNA by using a high-capacity cDNA reverse transcription kit (Applied Biosystems, Cat.:4368814). The primer sequences are shown as follows: *Gapdh,* 5’- AGGTCGGTGTGAACGGATTTG-3’ (sense) and 5’-TGTAGACCATGTAGTTGAG

GTCA-3’ (antisense); SARS-CoV-2 primer1 (*ORF1ab*): 5’-CCCTGTGGGTTTTACA

CTTAA-3’ (sense) and 5’-ACGATTGTGCATCAGCTGA-3’ (antisense); SARS-CoV-2 primer2 (*N*): 5’-GGGGAACTTCTCCTGCTAGAAT-3’ (sense) and 5’-CAGACATT

TTGCTCTCAAGCTG-3’ (antisense); *TNF-α*, 5’- CCACGTCGTAGCAAACCAC-3’ (sense) and 5’-TTGTCCCTTGAAGAGAACCTG-3’ (antisense); *IL-1β*, 5’- GCAACT

GTTCCTGAACTCAACT -3’ (sense) and 5’- ATCTTTTGGGGTCCGTCAACT -3’ (antisense); *IL-6*, 5’-TAGTCCTTCCTACCCCAATTTCC -3’ (sense)and 5’- TTGGTC

CTTAGCCACTCCTTC -3’ (antisense); C*tsl*, 5’-ATCAAACCTTTAgTgCAgAgTg

g-3’ (sense) and 5’-CTgTATTCCCCgTTgTgTAgC-3’ (antisense).

**Immunofluorescence staining**

Cells fixed and permeabilized for 5 min with 0.2% Triton X-100 (Sigma, Cat.: X-100). After washed with PBS, cells were blocked with 5% BSA and incubated with anti-NP antibody (Abcam, Cat. Ab273434, 1:200) at 4 ℃ overnight. Subsequently, cells were washed and incubated with secondary antibody for 1 h. Finally, cells were counterstained with DAPI (Sigma, Cat.: D9542) and mounted for confocal analysis. The intensity of immunofluorescence was analyzed by Image J 9.0 software.

***Ctsl* knockdown by siRNA**

AMs isolated from ICR mice were transfected with 20 pmol siRNA (RiboBio, China) against *Ctsl mRNA or* negative control siRNA (NC) using 4D-Nucleofector^TM^ X Kit (Lonza, Switzerland). The target sequences of siRNAs are shown as follows: siRNA1, CTACTATGAACCCAACTGT; siRNA2, GGCTATGAAGGAACAGATT.

**Animal experiments**

Rhesus macaques (3 to 4 kg, 3 to 4 years of age) were infected with SARS-CoV-2 (10^6^ TCID_50_) by intratracheal administration. After 7 days of treatment, macaques were euthanized and lung tissues were collected for histological staining.

**Statistical Analysis**

All experiments were performed at least three times. Results are expressed as mean ± SD as indicated and analyzed by one-way ANOVA followed by Bonferroni’s test. P< 0.05 was considered statistically significant. The analysis was conducted using the Graphpad 8.0 software.
